# Kappa Opioid Receptor on Pulmonary Macrophages and Immune Function

**DOI:** 10.31480/2330-4871/117

**Published:** 2020-02-20

**Authors:** Si Zeng, Yinbo Zhong, Jie Xiao, Jiafu Ji, Jin Xi, Xinchuan Wei, Renyu Liu

**Affiliations:** 1 Department of Anesthesiology, Sichuan Academy of Medical Science & Sichuan Provincial People’s Hospital, Electronic Science and Technology University, Chengdu, China; 2 Department of Anesthesiology and Critical Care, Perelman School of Medicine at the University of Pennsylvania, Philadelphia, PA, USA; 3 Department of Anesthesiology, The Second Affiliated Hospital Zhejiang University School of Medicine, Hangzhou, China; 4 Department of Anesthesiology, Renji Hospital, School of Medicine, Shanghai Jiaotong University, Shanghai, China; 5 Department of Anesthesiology, Affiliated Hospital of Shandong University of Traditional Chinese Medicine, Jinan, China

**Keywords:** Alveolar macrophages, Kappa opioid agonist, Salvinorin A, Pulmonary inflammation, Lipopolysaccharide, U50488

## Abstract

**Background:**

Respiratory failure significantly increases mortality in critically ill patients. While opioids are often used during the perioperative period and in critically ill situations, little is known about how opioids are involved in pulmonary immune function and the inflammatory response. There is currently no clear information on the role of the kappa opioid receptor (KOR) in pulmonary inflammation. Here we evaluate whether KORs are involved in the modulation of lung macrophages by the use of selective KOR agonists in lipopolysaccharide (LPS) activated alveolar macrophages.

**Method:**

The inflammatory response in rat NR8383 macrophages was induced by stimulation with LPS (100 ng/ml) at different time-points. The effects of the KOR agonists Salvinorin A (SA) and U50488 on inflammatory factors such as nitrite, TNF-α, IL-1β, iNOS and COX-2 were investigated. Nor-binaltorphimine, a selective KOR antagonist, was used to investigate the specific role of KOR.

**Results:**

Stimulation of NR8383 cells with LPS (100 ng/ml) significantly increased the level of TNF-α at 1h, 2h and 6h compared to un-stimulated cells. SA attenuated the inflammatory response by reducing the levels of TNF-α and IL-1β after LPS treatment. SA co-treatment reduced the elevated levels of NO induced by LPS and also alleviated the over-expression of iNOS and COX-2 within 2 hours after LPS activation, and such effects can be partially blocked by KOR antagonist, nor-binaltorphimine. Similar results from U50488 were observed.

**Conclusion:**

Our results indicate that KORs may play a critical role in the modulation of the pulmonary inflammatory process by their activation in macrophages. Selective KOR agonists exert their anti-inflammatory effects acutely on lung macrophages, within 1–2 hours of LPS-stimulated inflammation *in vitro*.

## Introduction

The lungs are critically important for oxygen exchange. The lungs generally are the first organs to fail in patients who are critically ill, especially when there is physiological and pathological response to an inflammatory process. While opioids are widely used in the perioperative period, when patients are critically ill, little is known about their role in pulmonary immune function and the process of inflammatory response. Among the different subtypes of opioid receptors: μ (mu), δ (delta), and κ (kappa), it is the kappa opioid receptors (KOR) that have been considered as potential therapeutic targets for disorders involving inflammatory processes, since they play a vital role in the treatment of complex central nervous system (CNS) and inflammatory disorders [1–5]. It has been demonstrated that KORs can modulate innate immune response in the teleost Channa punctatus by mechanisms involving endogenous dynorphin [6].

Alveolar macrophages (AMs), the first line of defense against pathogenic microorganisms and lung injury, are the main immune cells of the respiratory tract and the initial responders to foreign objects and trauma [7,8]. AMs can produce and release a variety of pro- and anti-inflammatory cytokines to modulate the immune response. While it is known that pro-inflammatory cytokines can up-regulate KOR expression in macrophages, [9], it is unclear whether KORs play a critical role in AM induced inflammatory responses in lungs.

There are two chemical categories of KOR agonists the traditional opioid KOR agonists and the novel non-opioid KOR agonists which have no nitrogen atoms in their molecular structure. The typical representative compound for the latter is salvinorin A (SA), the first highly selective non-opioid kappa agonist discovered, and which is derived from the plant Salvia divinorum. SA is considered to be the most potent naturally occurring KOR agonist [10,11]. Our previous studies have shown that a KOR agonist can protect the brain from ischemic/reperfusion and hypoxic injury [3,5,12,13]. Although there are numerous reports on the central nervous system effects of SA, little is known about its anti-inflammatory properties in the pulmonary system. It has been shown that SA can reduce lipopolysaccharide (LPS)-induced nitrite, inducible nitric oxide synthase (iNOS) and tumor necrosis factor-α (TNF-α) production in murine peritoneal macrophages [14]. Furthermore, SA inhibited leukotriene synthesis in experimental models of inflammation [15]. U50488 is also a highly selective KOR agonist and it has been shown to modulate macrophage function through KORs [16]. These studies suggest that KORs may play a significant role in the process of inflammatory response in the pulmonary system by their activation in macrophages.

In the present study, we investigated whether KORs are involved in the modulation of lung macrophages (NR8383 cells) by using two highly selective KOR agonists (SA and U50488) in LPS-activated rat alveolar macrophages.

## Materials and Methods

SA (purity ≥ 98%) was obtained from Apple Pharms (Asheville, NC). U-50488 hydrochloride (U50488; purity > 99%) and nor-Binaltorphimine dihydrochloride (Nor-Bin; purity, > 98%) were purchased from Tocris Bioscience (Minneapolis, MN). D-methyl-sulfoxide (DMSO) was used as a solvent in saline solution (0.01% final concentration of DMSO). All other compounds were obtained from Sigma-Aldrich (St. Louis, MO).

### Stimulation and treatment of NR8383 macrophages

NR8383 cells (CRL-2192), a rat alveolar macrophage cell line (ATCC, Rockville, USA), were maintained in plastic tissue culture flasks in Hans F-12 medium containing 15% heat inactivated fetal bovine serum (FBS), 100 U/ml penicillin, 100 pg/ml streptomycin, 2 mM L-glutamine (Biowhittacker, Verviers, Belgium) and 1.5 g/l sodium bicarbonate. Cells were cultured at 37 °C in a humidified, 5% CO_2_ incubator. The medium was routinely changed twice weekly.

Cells were stimulated with 100 ng/ml LPS (Sigma, St. Louis, USA) in the presence or absence of various concentrations of freshly prepared SA (10^−6^–10^−15^ M) or U50488 (10^−9^–10^−11^ M) for 1h, 2h, 4h, 6h and 24h with or without nor-binaltorphimine (30 nM) pretreatment 30 min before LPS. Cells and supernatants were collected at various time points and stored at −80 °C for various measurements described below.

### LDH cytotoxicity assays

Release of lactate dehydrogenase (LDH) into the media was quantified using an LDH Cytotoxicity Assay Kit (The Thermo Scientific Pierce™) according to the manufacturer’s instructions.

### Nitrite measurement

NR8383 cells were seeded into 96-well plates at 1 × 10^5^ cells per well and treated with LPS. The activated cells were exposed to SA in the presence or absence of Nor-binaltorphimine (Nor-Bin) for 2h and 4h. Nitrites were measured in the culture medium using the Griess Reagent Kit (Molecular Probes, USA) according to the manufacturer’s instructions [17]. Samples were measured at 548 nm using Synergy™ H1 microplate reader (BioTek, Winooski, VT).

### Western blot

Cells (passage number 8) in six-well culture plates were washed twice with PBS and lysed on ice with 50 mM Tris-HCl buffer (pH 6.8) containing 20% glycerol, 1% sodium dodecyl sulfate (SDS) and protease inhibitor cocktail (Sigma, St. Louis, MO). Equal amounts of protein lysates (30 μg) were subjected to 10% SDS-PAGE and transferred onto PVDF membranes (Bio-Rad Laboratories, Hercules, CA, USA). Membranes were blocked in Tris buffer saline containing Tween-20 (TBST) with 5% BSA for 2 hours at room temperature and incubated overnight at 4 °C with monoclonal antibodies against iNOS 2 (Santa Cruz Biotechnology, Dallas, TX, sc-615, Lot # I2815 and COX-2 (Santa Cruz Biotechnology, Dallas, TX, sc-7951, Lot # D1015). Proteins were then detected with secondary goat anti-mouse IgG-HRP (Santa Cruz Biotechnology, Dallas, TX) for 1 hour at room temperature and developed using the ECL Western Blotting Substrate (Thermo Fisher Scientific). β-Actin was used as a loading control. Images were quantified with Image J 1.49v software (National Institutes of Health, Bethesda, MD).

### Determination of cytokines

Cell culture supernatants were collected and the levels of TNF-α and IL-1β were measured using ELISA kits (Thermo scientific, USA) according to the manufacturer’s instructions.

### Statistical analysis

All data were expressed as mean ± standard error of the mean (SEM). The statistical analysis was carried out using Graph-Pad Prism 6 software (GraphPad Software, Inc., La Jolla, CA). Statistical analysis was performed using One-way analysis of variance (ANOVA) followed by Tukey’s test for multiple comparisons. A value of P < 0.05 was considered statistically significant.

## Results

### LPS, SA or U50488 have no cytotoxicity in alveolar macrophage cells at tested conditions

**Figure 1:**
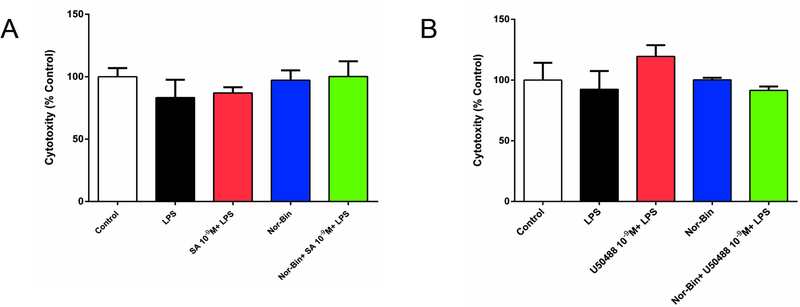
Lipopolysaccharide, salvinorin A (SA) or U50488 did not induce rat alveolar macrophage cytotoxicity. Rat alveolar macrophages (AMs) were exposed to lipopolysaccharide (LPS), SA (10^−9^ M), U50488 (10^−9^ M) or nor-binaltorphimine (Nor-Bin, 30 nM) for 2h. Cell damage was determined by lactate dehydrogenase (LDH) release assay. Compared with vehicle (DMSO) controls, LPS alone, LPS with SA or U50488, Nor-Bin, co-treatment of LPS, SA or U50488 and Nor-Bin did not induce cell damage after 2h exposure. All data are given as mean ± SEM from at least three separate experiments and analyzed by one-way ANOVA followed by Tukey’s multiple comparisons test.

Compared with vehicle controls, LPS alone (100 ng/ml) or co-incubated with SA (10^−9^ M), U50488 (10^−9^ M) or Nor-Bin (30 nM) did not cause cell damage after 2h exposure (Figure 1A and Figure 1B).

### KOR agonists inhibited TNF-α production in LPS-activated alveolar macrophages

**Figure 2:**
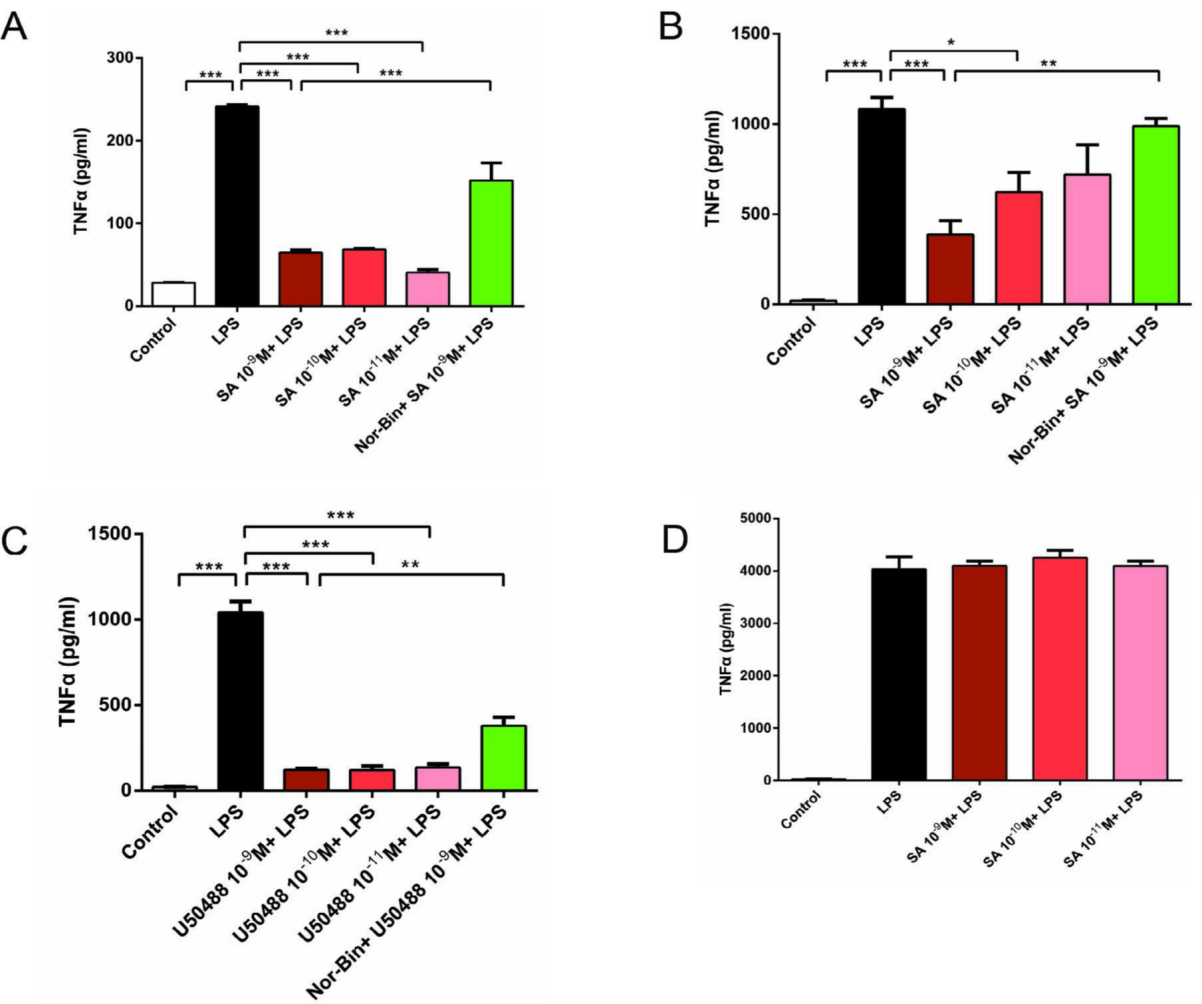
Selective kappa opioid agonists inhibit lipopolysaccharide (LPS)- induced elevation of tumor necrosis factor-α (TNF-α) in alveolar macrophages. Rat alveolar macrophage cell (AMs) were exposed to lipopolysaccharide (LPS), salvinorin A (SA), U50488 or nor-binaltorphimine (Nor-Bin, 30 nM) for 2h and 6h. SA significantly decreased the TNF-α level induced by LPS at 1h **(A)** and 2h **(B)** after exposure, but not at 6h **(D)**. These anti-inflammatory effects were inhibited by pretreatment of Nor-Bin. U50488 significantly decreased the TNF-α level induced by LPS at 2h, which was inhibited by pretreatment of Nor-Bin **(C)**. All data are given as mean ± SEM from at least three separate experiments and analyzed by one-way ANOVA followed by Tukey’s multiple comparisons test. ^**^P < 0.01, ^***^P < 0.001 as indicated.

Stimulation of NR8383 cells with LPS (100 ng/ml) significantly increased the level of TNF-α at 1h, 2h and 6h compared to un-stimulated cells. Co-treatment of LPS-stimulated AMs with SA (10^−9^ M, 10^−10^ M and 10^−11^ M for 1 h; 10^−9^ M and 10^−10^ M for 2h) inhibited TNF-α production (Figure 2A and Figure 2B). However, inhibition was not prominent after 6h of stimulations (Figure 2D). Pretreatment with Nor-Bin (30 nM) blocked the anti-inflammatory effects of SA (10^−9^ M; Figure 2A and Figure 2B). U50488 (10^−9^ M) significantly decreased the TNF-α level induced by LPS at 2 h, and this effect was inhibited by pre-treatment with Nor-Bin (10^−9^ M; Figure 2C).

### SA inhibits production of nitrites in LPS-activated alveolar macrophages via KOR

**Figure 3:**
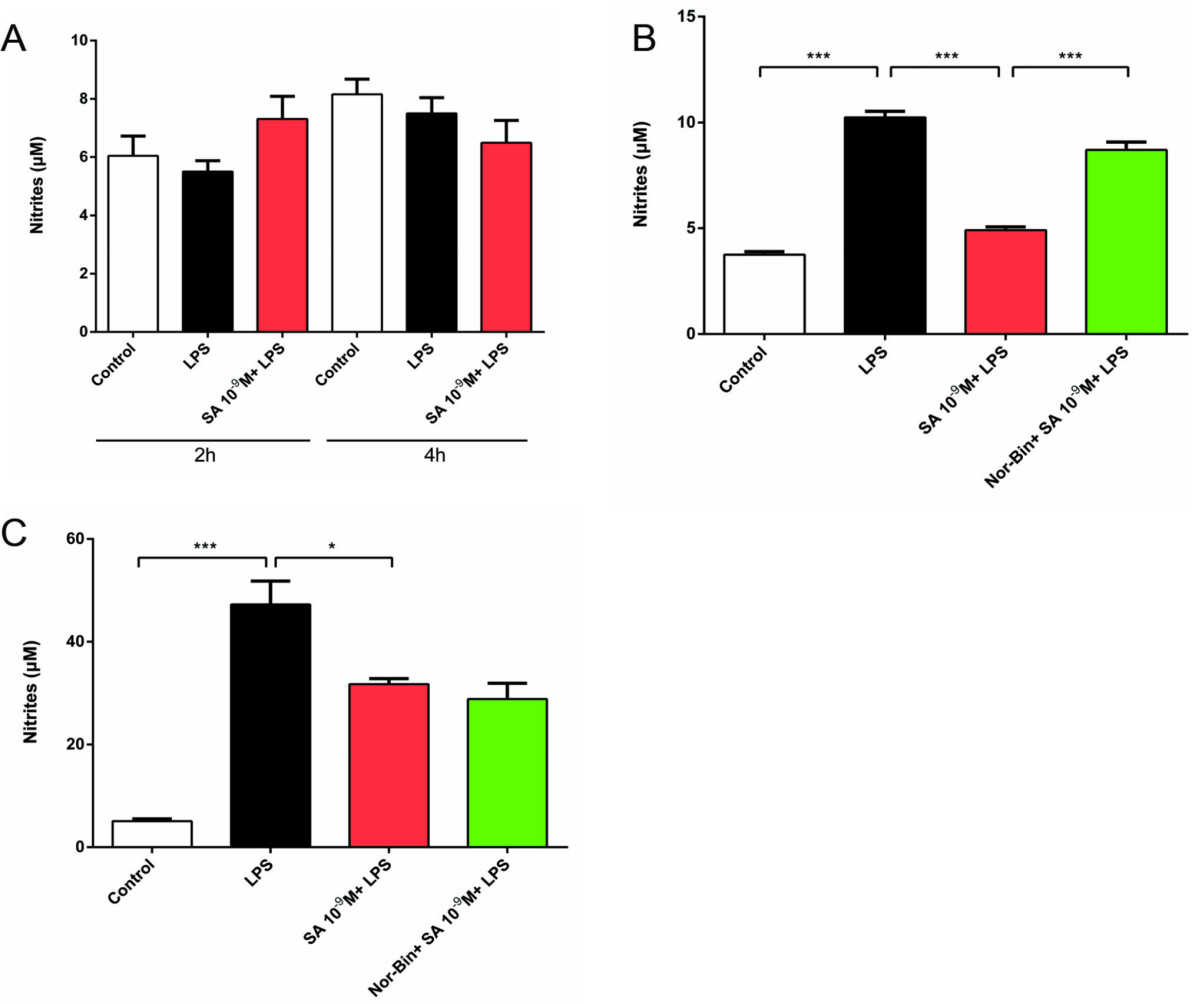
Selective kappa opioid agonists inhibit lipopolysaccharide-induced elevation of nitrites in alveolar macrophages. Rat alveolar macrophage cells (AMs) were exposed to lipopolysaccharide (LPS), salvinorin A (SA, 10^−9^ M) or nor-binaltorphimine (Nor-Bin, 30 nM) for 2h, 4h, 6h and 24h. LPS exposure increased nitrites level only at 6h and 24h, but not 2h and 4h **(A)**. SA decreased the nitrites level induced by LPS at 6h, which was inhibited by pretreatment of Nor-Bin **(B)**. SA decreased the nitrites level induced by LPS at 24h, which was not inhibited by pretreatment of Nor-Bin **(C)**. All data are given as mean ± SEM from at least three separate experiments and analyzed by one-way ANOVA followed by Tukey’s multiple comparisons test. *P < 0.05, ^**^P < 0.01, ^***^P < 0.001 as indicated.

LPS (100 ng/ml) exposure increased the level of nitrites for 2h and 4h (Figure 3A). Stimulation of rat AMs with LPS (100 ng/ml) induced a significant increase of nitrites for 6h and 24 (Figure 3B and Figure 3C). Co-treatment of LPS-stimulated AMs with SA (10^−9^ M) suppressed the nitrite levels at 6h, and this effect is blocked by pre-treating with Nor-Bin (10^−9^ M; Figure 3B). After co-treatment of AM with SA for 24h, nitrite production significantly decreased. In this case, nitrite production was not blocked by pretreatment with Nor-Bin (10^−9^ M; Figure 3C).

### KOR agonists inhibit IL-1β production in LPS-activated alveolar macrophages

**Figure 4:**
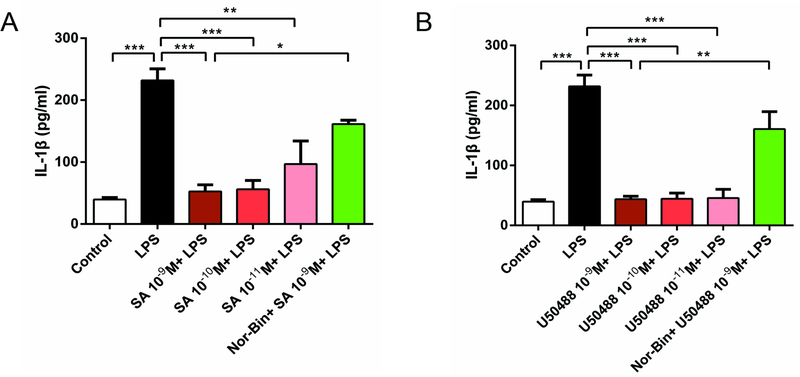
Selective kappa opioid agonists inhibit lipopolysaccharide-induced elevation of Interleukin 1β (IL-1β) in alveolar macrophages. Rat alveolar macrophage cells (AMs) were exposed to lipopolysaccharide (LPS), salvinorin A (SA), U50488 or nor-binaltorphimine (Nor-Bin, 30 nM) for 2h. Both SA **(A)** and U50488 **(B)** decreased the IL-1β level induced by LPS at 2h, which was inhibited by pretreatment of Nor-Bin. All data are given as mean ± SEM from at least three separate experiments and analyzed by one-way ANOVA followed by Tukey’s multiple comparisons test. *P < 0.05, ^**^P < 0.01, ^***^P < 0.001 as indicated.

Co-treatment with LPS and SA (10^−9^ M, 10^−10^ M and 10^−11^ M, Figure 4A) or U50488 (10^−9^ M, 10^−10^ M and 10^−11^ M, Figure 4B) suppressed IL-1β production induced by LPS (100 ng/ml) for 2h. This effect was blocked by pre-treatment with Nor-Bin (10^−9^ M; Figure 4A and Figure 4B), indicating the specificity of KOR involvement.

### KOR agonists inhibit iNOS and COX-2 expression in activated alveolar macrophages

**Figure 5:**
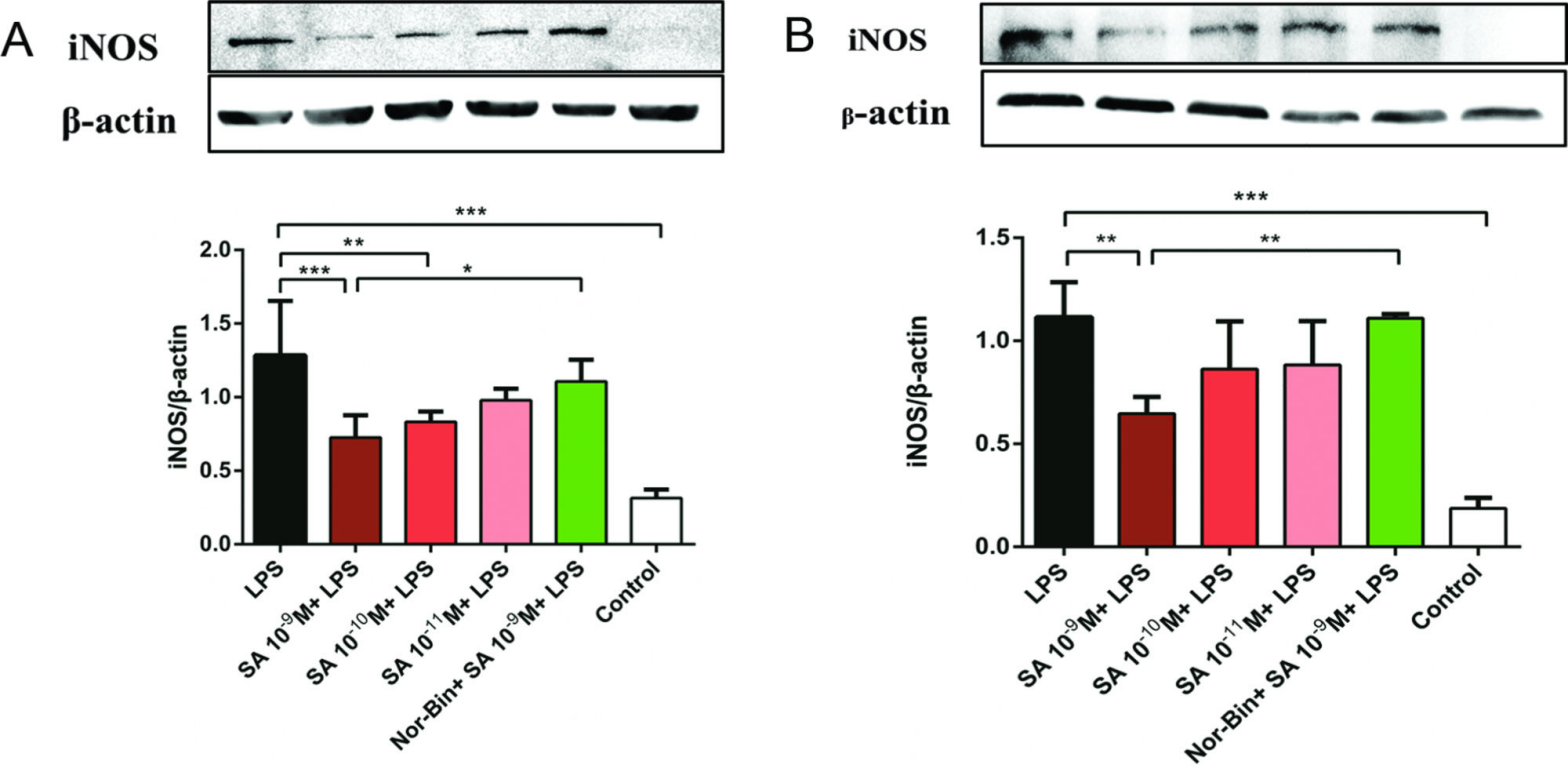
Selective kappa opioid agonists inhibit lipopolysaccharide-induced elevation of inducible nitric oxide synthase in alveolar macrophages. Quantitative analysis of immunoblotting of inducible nitric oxide synthase (iNOS) from rat alveolar macrophage cells cultured with lipopolysaccharide (LPS), salvinorin A (SA) or nor-binaltorphimine (Nor-Bin, 30 nM) for 2h **(A)** and 4h **(B)**. iNOS/β-actin ratio showed that LPS increased level of iNOS at exposure duration of 2h (n = 5) and 4h (n = 4), which could be inhibited by co-treatment of SA 10^−9^ M, 10^−10^ M at 2h and SA 10^−9^ M at 4h. These anti-inflammatory effects of SA 10^−9^ M were alleviated by pretreatment of Nor-Bin. Data was analyzed by one-way ANOVA followed by Tukey’s multiple comparison tests. *P < 0.05, ^**^P < 0.01, ^***^P < 0.001 compared with corresponding time control or indicated.

**Figure 6:**
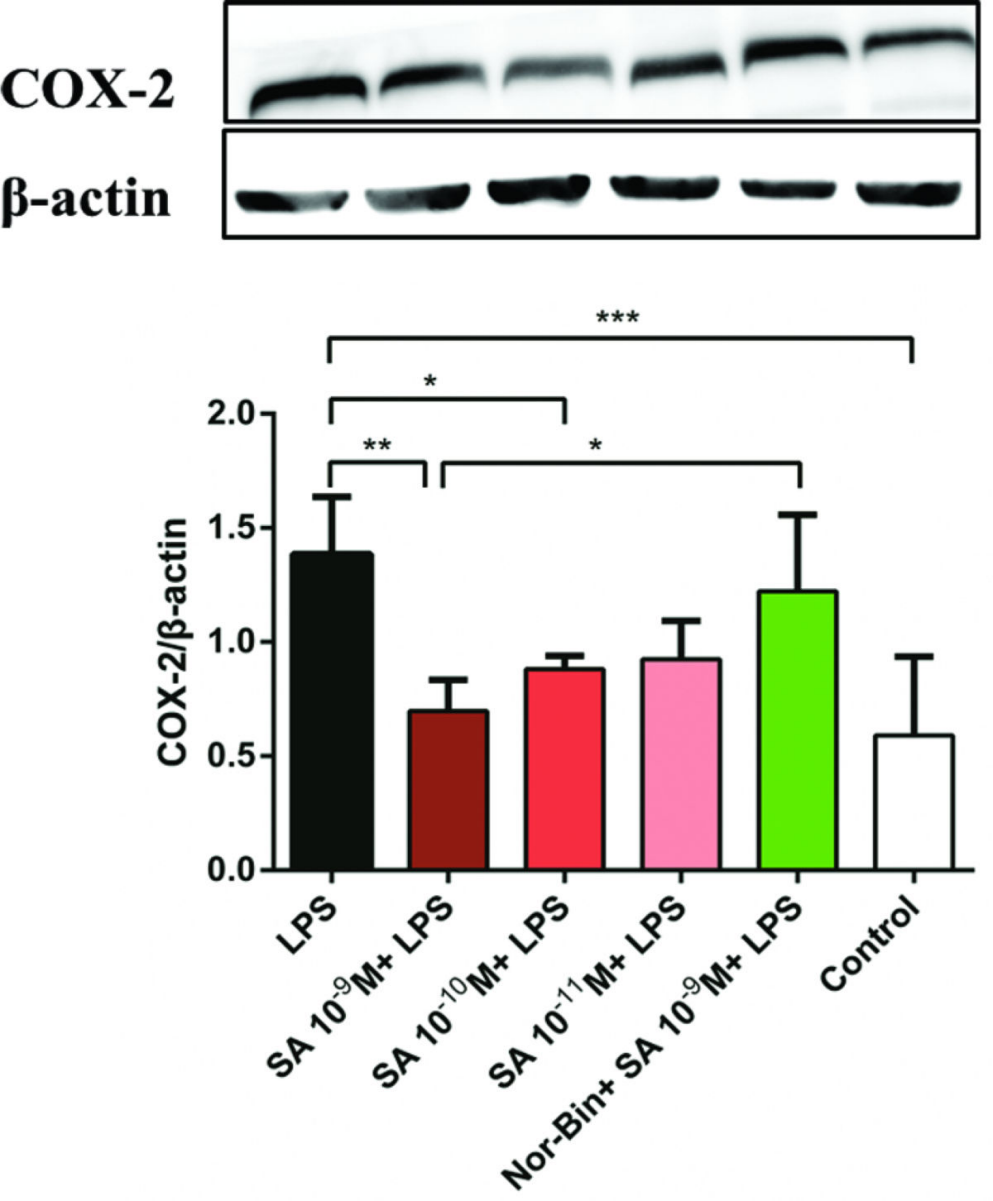
Selective kappa opioid agonists inhibit lipopolysaccharide-induced elevation of cyclooxygenase-2 (COX-2) in alveolar macrophages. Quantitative immunoblotting analysis of COX-2 from rat alveolar macrophage cells cultured with lipopolysaccharide (LPS), salvinorin A (SA) or nor-binaltorphimine (Nor-Bin, 30 nM) for 2h **(A)**. COX-2/β-actin ratio showed that SA 10^−9^ M, 10^−10^ M decrease the over-expression of LPS-induced levels of COX-2 at stimulation time of 2h (n = 5), which was alleviated by pretreatment of Nor-Bin. Data was analyzed by one-way ANOVA followed by Tukey’s multiple comparison tests. *P < 0.05, ^**^P < 0.01, ^***^P < 0.001 compared with corresponding time control.

Overexpression of iNOS was induced by LPS (100 ng/ml) stimulation for 2h and 4h (Figure 5A and Figure 5B). Co-treatment with SA (10^−9^ M and 10^−10^ M for 2h, Figure 5A; 10^−9^ M for 4h, Figure 5B) significantly inhibited the iNOS overexpression in LPS-induced AMs. Pretreatment with Nor-Bin (30 nM) blocked the inhibitory effects at 2h (Figure 5A) and 4h (Figure 5B) of SA (10^−9^ M). SA (10^−9^ M and 10^−10^ M) inhibited the overexpression of LPS-induced COX-2 at 2h, which was blocked by pretreatment of Nor-Bin (10^−9^ M; Figure 6).

## Discussion

Alveolar macrophages protect the lung acting as a first line host defense by absorbing foreign particles by phagocytosis, which is a crucial mechanism in innate and adaptive immunity. Once AMs are activated (in this case, stimulated with LPS), various inflammatory mediators are released. These inflammatory mediators can be inhibited by KOR agonists during the acute phase, as demonstrated in this study.

Neither SA (10^−6^ −10^−15^ M) nor U50488 (10^−7^-10^−9^ M) was cytotoxic to AMs. Consistent with this finding, SA did not exhibit acute or chronic cytotoxicity in animal models [18]. However, dose-dependent cytotoxic effects of SA have been reported in some cell lines *in vitro* [19].

In the present study, SA reduced the production of COX-2, a key enzyme involved in the inflammatory response that catalyzes the biosynthesis of prostaglandins [20]. Previous studies indicated that SA inhibited leukotriene synthesis with high efficiency in rat peritoneal macrophages, in Zymosan-induced peritonitis in mice, and in carrageenan-induced pleurisy in rats [15]. While SA demonstrated strong anti-inflammatory effects in peritoneal macrophages, it did not affect the induction of COX-2 and IL-1β [14]. However, in this study, KOR agonists decreased IL-1β as well as COX-2 levels, especially within 2 hours of LPS stimulation. This indicates that there may be a fundamental difference in the process of inflammation in the lung as compared to the gastro-intestinal system and KOR agonists generate anti-inflammatory effects in different organ systems. This data also shows that there is some linkage between IL-1β and COX-2, which has been demonstrated in the inflammatory process in the central nervous system [21]. It is possible that SA may act on the COX-2 pathways via KORs and reduce PEG2 levels. Further study of this mechanism is needed [22].

Nitric oxide (NO) has been identified as a potent molecule involved in important regulatory functions and toxicity effects [23]. The arginine-dependent enzyme inducible nitic oxide synthase iNOS increases during autoimmune reactivity conditions. Our results demonstrate that SA treatment reduced iNOS production within 2 hours of LPS treatment. Consistent with this finding the KOR agonists down-regulated nitrite levels, which are the stable metabolites of NO, in the supernatant of LPS-induced AMs, at 6 hours and 24 hours. Interestingly, the selective KOR antagonist could partially reverse the anti-inflammatory effects of SA in AMs at 6 hours, but not at 24 hours. This indicates that the down regulation of nitrite levels at different time points may be modulated by distinct signaling pathways, and KORs may only play a critical role in the acute phase of the inflammatory process.

LPS increases the levels of TNF-α and IL-1β by interaction with Toll-like receptor 4 (TLR-4) [24,25]. The levels of TNF-α and IL-1β are regulated by a nuclear factor-kappa B (NF-κB) [26,27]. While these cytokines are key elements of the innate immune response, their over-expression may lead to the acute phase inflammation of endotoxemia. Therefore, regulating the expression of these inflammatory mediators is essential for managing inflammatory disorders. According to a previous study [28], SA reduced TNF-α and IL-10 (but not IL-1β) levels in LPS-stimulated peritoneal macrophages [14]. Our study showed that both, SA and U50488 inhibited the expression of the pro-inflammatory cytokines TNF-α and IL-1β in LPS-stimulated AMs. This observation further confirms that KOR agonists possess anti-inflammatory properties in LPS-stimulated AMs. However, further investigations are needed to elucidate whether there are interactions between KORs and TLR 4 mediated signaling pathways, such as NF-κB, MAPK and others involved in inflammatory processes.

**Figure 7:**
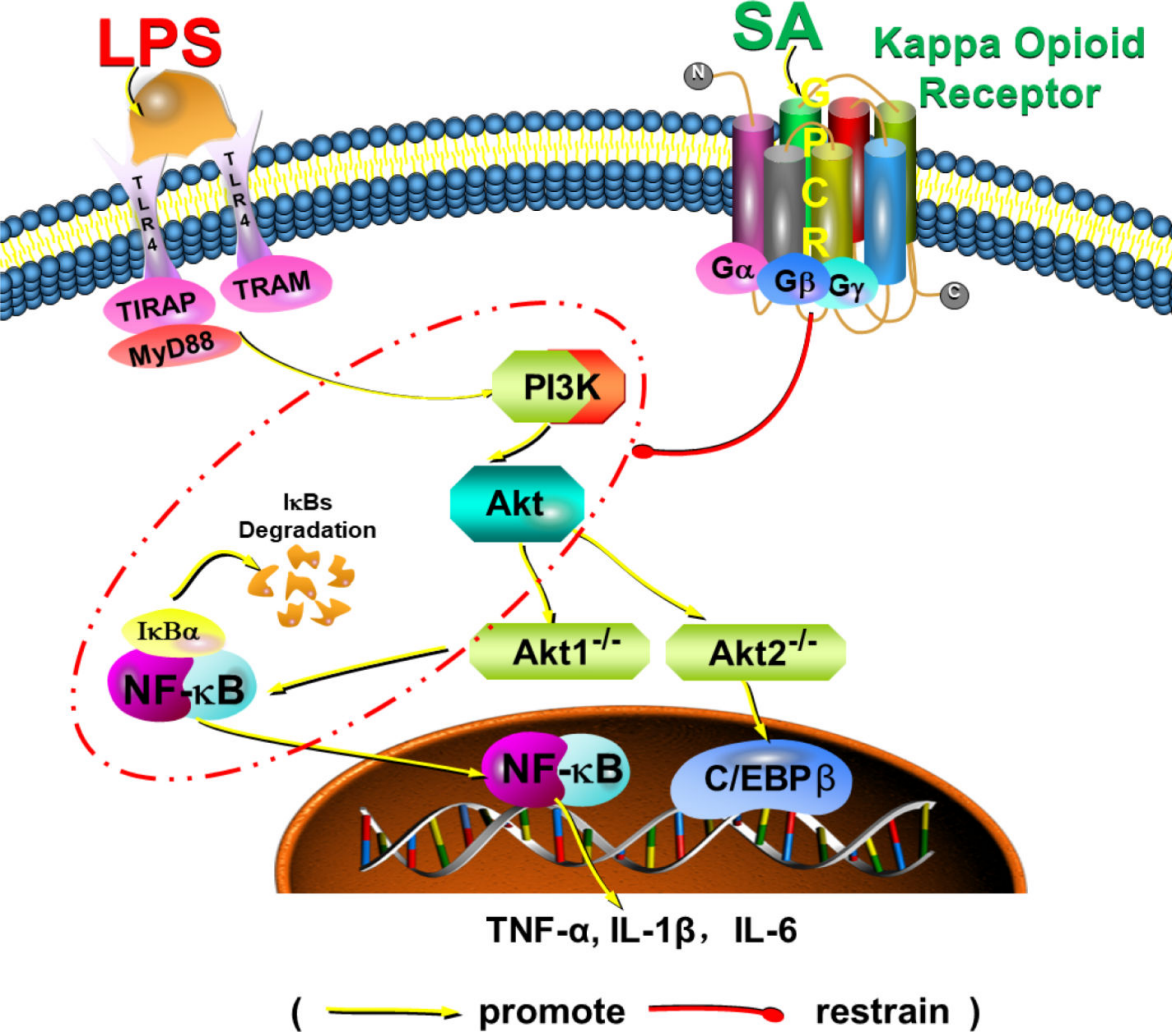
A hypothetical schematic graph of KOR interaction and underlying cytokine production cascade to explain the possible underlying mechanisms. SA, salvinorin A; LPS, lipopolysaccharide; TLR, Toll-like receptor 4; GPCR, G coupled protein receptor; TNF, Tumor necrosis factor; IL, Interleukin.

Both TLR4 and KOR are expressed in macrophages. To date, the mechanism of KOR inhibition of LPS-induced TLR4 signaling pathway is not clear. The crosstalk between TLR4 and KOR pathway may play a key role in modulation of immune system. In our study, SA and U50488 inhibited the expression of pro-inflammatory cytokines TNF-α and IL-1β in LPS-stimulated AMs. LPS activate TLR4 signaling pathway and lead to expression of Nuclear factor-κB (NF-κB) and release of proinflammatory cytokines such as TNF-α, IL-1 and IL-6 [24,25]. NF-κB is an important transcription factor regulating macrophage induced inflammation and immune response [26,27], and is a key signal factor in PI3K/Akt signaling pathway [29,30]. Thus, whether this negative crosstalk might locate in the upstream of PI3k/Akt/NF-κB other signals still need further study. Here, we presented a hypothetical schematic graph of KOR interaction and underlying cytokine production cascade as shown in Figure 7.

Interestingly, we found that although SA suppressed the production of TNF-α, IL-1β, iNOS and COX-2 within 2 hours in LPS-stimulated alveolar macrophages, it failed to exert these effects after 4 hours of stimulation, suggesting the involvement of KORs is in the acute phase only. Further studies are needed to investigate the mechanism of such phenomena.

The limitation of our experiment is that the results need to be verified in an animal lung injury model such as LPS-instilled lung injury model or ventilator-induced lung injury model in the future. The main reason for using rat NR8383 macrophage cell line in this study is due to the favorable stability of the cell line. These results could potentially be confirmed using the freshly collected murine or rat alveolar macrophages despite the results could potentially be affected by other factors before the cells are harvested *in vivo*.

In summary, this study demonstrates anti-inflammatory properties of selective kappa opioid agonists, including non-opioid SA and nitrogenous U50488, in alveolar macrophages. KOR agonists reduced LPS-induced iNOS, COX-2, TNF-α, and IL-1β over-expression in alveolar macrophages within 2 hours after LPS activation, and such effects can be partially blocked by KOR antagonist, suggesting KOR might play critical role in modulating the acute phase of pulmonary inflammation via macrophages.
